# Optimizing the InterRAI Assessment Tool in Care Planning Processes for Long-Term Residents: A Scoping Review

**DOI:** 10.1177/10547738211020373

**Published:** 2021-05-30

**Authors:** Steve Iduye, Tracie Risling, Shelley McKibbon, Damilola Iduye

**Affiliations:** 1 University of Saskatchewan, Saskatoon, Saskatchewan, Canada; 2 Dalhousie University, Halifax, Nova Scotia, Canada

**Keywords:** interRAI, standardized data, long-term care, care planning, health outcomes

## Abstract

The aim of this review was to chart and report on existing literature that discusses how the interRAI assessment tool drives care-planning processes for residents in long-term-care settings. This scoping review was informed by the Joanna Briggs Institute guidelines for scoping reviews and the Preferred Reporting Items for Systematic Reviews and Meta-Analyses extension for Scoping Reviews guideline. Relevant studies were obtained from databases search of CINAHL (EBSCO), MEDLINE (Ovid), PsycINFO (EBSCO), Academic Search Premier (EBSCO), Embase (Elsevier), ProQuest Nursing and Allied Health Database (ProQuest), Sociological Abstracts (ProQuest), and Social Services Abstracts (ProQuest). Of the 17 included studies, five (29.4%) addressed interRAI’s minimum dataset component as a clinical data-collection tool; five (29.4%) addressed interRAI’s assessment scales and its clinical-assessment protocols as viable health-assessment tools; four (23.5%) considered interRAI’s assessment scales in terms of whether this tool is capable of predicting residents’ health risks; one (5.9%) addressed the effects of interRAI’s care plans on residents’ health outcomes; and the remaining two studies (11.8%) used interRAI’s quality-indicator function for both the performance of and improvements in the quality of care. The scoping review finds that there is no substantial evidence that supports the implementation of interRAI care plans for consistent health outcomes.

## Introduction

The world’s senior population will reach 2.1 billion by 2050 (United Nations, Department of Economic and Social Affairs, Population Division, 2017). In Canada, adults aged 65 years and over will represent between 23% and 25% of the population by 2036 (Statistics Canada, 2015). In 2016, Canada’s older demographic consisted of 5.9 million adults aged 65 and above; 93.2% of these seniors lived in private houses, apartments, or moveable dwellings, while 6.8% lived in senior citizens’ residences and long-term-care facilities (LTCFs) (Government of Canada, 2019). Statistics Canada projects that this population of seniors will continue to increase, across the country, over the next few decades (Statistics Canada, 2020). However, there are increasing concerns regarding the quality of care that will be provided to elderly residents as their population continues to grow. Across Canada, the provision of quality care to seniors in long-term care residences has suffered from poor government funding, neglect, and the mishandling of staff as human capital, all of which pervade long-term care and its administration. In most LTCFs, there is high turnover among registered nurses (RNs) and retention is difficult; several attempts to address the situation have not produced positive results (Collier & Harrington, 2008). Moving from traditional care planning that pays little or no attention to the needs or preferences of residents in long-term care (LTC) to care planning that focuses on how these residents can make contributions to their own care, with proper documentation for all clinical staff to follow, is a key quality indicator (Social Care Institute for Excellence, 2017).

Long-term care facilities that provide 24-hour support are often called nursing homes, long-term care facilities, residential care centers or seniors’ residences (Macdonald et al., 2020). Care planning consists of negotiations and agreements between care providers and residents to develop relevant health plans throughout the interrelated processes of performing health assessments, formulating care plans, and implementing and evaluating the care provided (Burt et al., 2014). To promote quality care delivery in LTCs, one approach recommended in the literature is clinical information management systems. The international resident assessment instrument (interRAI) is one such tool that has received support for implementation in Canada. The interRAI is a data-driven application that nurses and other clinicians use to collect clinical data upon a resident’s admission, and again quarterly and annually, so as to generate plans that inform the care that is administered to a particular LTC resident (Armstrong et al., 2017; Hutchinson et al., 2010). Evidence suggests that interRAI is a reliable and valid assessment tool in chronic disease management related to LTC residents (Chou et al., 2001; Kim et al., 2015). However, the degree to which interRAI care plans drive better outcomes in resident care processes is under-investigated (Bott et al., 2007; Daly et al., 2002). The few studies that examined interRAI’s application for better health outcomes found inconsistent use of care plans and, consequently poorer health outcomes for residents (Colón-Emeric et al., 2006; Kontos et al., 2010; Schnelle et al., 2004). When using interRAI’s digital application in LTCs, nurses and other clinicians add residents’ clinical data, which then triggers a set of clinical-assessment protocols (CAPs) (Adams-Wendling et al., 2008). These CAPs are the problem areas in residents’ health that require care interventions and each resident’s care plan is informed by a set of CAPs from interRAI (Adams-Wendling et al., 2008; Dash et al., 2018).

A preliminary search of PUBMED, EMBASE, CINAHL, Social Services Abstract, Academic Premier, Nursing and Allied ProQuest, the Cochrane Database of Systematic Reviews, and the JBI Database of Systematic Reviews and Implementation Reports show no systematic or scoping review that investigates how the interRAI tool drives care-planning processes in LTCs. However, two current systematic reviews have evaluated the use of interRAI in home-care planning and health outcomes for frail older adults who are living outside LTC facilities. Mello et al. (2015) reviewed studies on interRAI-driven home care interventions for frail older people who are living in their homes within their communities. Their reported outcomes consider interRAI as a comprehensive health-assessment tool for this population. Moreover, Salahudeen and Nishtala (2019) evaluated studies that used the interRAI home-care instrument (interRAI HC) to examine outcomes for older people. In their review, the evidence shows that the instrument is valid; it also points to its utility as a quality indicator and evaluation tool for the health care of older adults who live in their community. In recent times, different suites of interRAI tools have been adapted to home and community care, mental health, and palliative and acute care. A recent upgraded version is specific to long-term care (interRAI-LTCF) (Adams-Wendling et al., 2008).

### Significance

Since little is known about how the interRAI care plans translate into consistent health outcomes for LTC residents (Adams-Wendling et al., 2008), it is useful to undertake a scoping review of the current studies on interRAI-driven care processes as they relate to older adults living in LTCs. This review will inform clinical practice and support the advancement of both research and policy priorities for chronic disease management in LTC. The study can help ascertain how the interRAI tool is used to generate applicable care plans and interventions and to evaluate care quality in residents’ health assessments. Improving the utility of interRAI-driven care plans for the residents will require increasing employment opportunities for frontline care providers. It will also provide an opportunity or platform for all care providers in LTCFs and for their residents and care representatives to collectively address the challenges associated with the care-planning processes and the implementation of these care plans. In this scoping review, we consider existing studies on the use of interRAI in care processes for LTC residents.

## Methods

### Search Strategy

The search strategy incorporated all study designs that could inform our knowledge regarding how the interRAI tool drives or could be used to coordinate care-planning processes in long-term-care facilities. Thus, the review objective is to chart and report the existing literature on how the interRAI tool drives residents’ care-planning processes in long-term-care settings. The Joanna Briggs Institute’s (JBI) three-step search strategy employed in this review includes (Peters et al., 2020) an initial search of the CINAHL database, using the keywords interRAI, long-term care, nursing homes, and care plan. This process was followed by an analysis of the textual words contained in the titles and abstracts of the relevant articles. This first step informed the further development of the search terms that were used in the databases searched. The second step was a search that used all of the identified keywords and index terms across each of the following databases: CINAHL (EBSCO), MEDLINE (Ovid), PsycINFO (EBSCO), Academic Search Premier (EBSCO), Embase (Elsevier), ProQuest Nursing and Allied Health Database (ProQuest), Sociological Abstracts (ProQuest), and Social Services Abstracts (ProQuest). In the last step, the reference lists of all of the selected studies were screened for additional relevant studies. The relevant studies published in any year, in the English language, were considered for inclusion (Table 1).

**Table 1.** Search Strategy (April 17th, 2020).

InterRAI	Health planning (HP)	Long term care (LTC)	Date run	Results
Keywords (EBSCO operators)				
interrai	(care N3 (activit* OR implement* OR plan* OR goal*))	(“long term care” OR “nursing home*” OR “care home*” OR residence* OR residential or ltc OR (extended W2 care) OR “longterm care”)		
CINAHL headings				
No heading	(MH “Patient Care Plans+”) OR (MH “Health and Welfare Planning+”)	(MH “Long Term Care”) OR (MH “Nursing Home Patients”) OR (MH “Nursing Homes+”)	17-Apr-20	138
PsycINFO headings				
No heading	(DE “Treatment Planning” OR DE “Caring Behaviors” OR DE “Discharge Planning” OR DE “Post-treatment Follow-up”) OR (DE “Case Management” OR DE “Discharge Planning”)	(DE “Nursing Homes”) OR (DE “Long Term Care”)	17-Apr-20	43
Acad search premier headings				
No heading	(DE “MEDICAL protocols” OR DE “ANTINEOPLASTIC combined chemotherapy protocols” OR DE “NURSING care plans” OR DE “PATIENT selection” OR DE “RADIOTHERAPY treatment planning”) OR (DE “MEDICAL case management” OR DE “HOSPITAL case management services” OR DE “TRANSFER of medical care”)	((DE “LONG term health care” OR DE “CHRONICALLY ill patient care” OR DE “CONTINUUM of care” OR DE “LONG-term care facilities” OR DE “NURSING home care”) OR (DE “NURSING care facilities” OR DE “DEMENTIA care units” OR DE “NURSING home chains” OR DE “TEACHING nursing homes”)) OR (DE “OLD age homes” OR DE “JEWISH old age homes”)	17-Apr-20	130
MEDLINE (Ovid) [MeSH terms]				
No heading	Exp patient care management/ or exp patient care planning/ or progressive patient care/	Exp Nursing Homes/ OR Long-Term Care/	17-Apr-20	199
Embase [Emtree headings]				
No heading	‘Long term care’/exp OR ‘long term care’ OR ‘nursing home*’ OR ‘care home*’ OR residence* OR residential OR ltc OR ‘extended care’/exp OR ‘extended care’ OR ‘longterm care’	‘Long term care’/exp OR ‘nursing home’/exp OR ‘nursing home patient’/exp	17-Apr-20	249
Nursing and allied health (ProQuest) headings				
No heading	MAINSUBJECT.EXACT (“Patient care planning”)	MAINSUBJECT.EXACT (“Long term health care”) OR MAINSUBJECT.EXACT (“Nursing homes”)	17-Apr-20	348
Sociological abstracts headings				
No heading	MAINSUBJECT.EXACT (“Health Planning”) OR MAINSUBJECT.EXACT (“Planning”)	MAINSUBJECT.EXACT (“Nursing Homes”) OR MAINSUBJECT.EXACT (“Long Term Care”)	17-Apr-20	36
Social services abstracts (same as Sociological abstracts headings)				
No heading	MAINSUBJECT.EXACT (“Health Planning”) OR MAINSUBJECT.EXACT (“Planning”)	MAINSUBJECT.EXACT (“Nursing Homes”) OR MAINSUBJECT.EXACT (“Long Term Care”)	17-Apr-20	40
Total in databases:	1184			
Duplicates removed:	569			
Total in covidence	615			

The core concepts of this review were the care processes used when collecting residents’ clinical data and how the collected data inform residents’ health assessments, the mutually agreed care plans for residents, and the implementation and evaluation of the care that was planned and provided through the use of the interRAI tool (Table 2). Included in the review were studies whose participants were aged 65 years or older who lived in nursing homes, long-term-care facilities, or long-term residential care or seniors’ residences. Excluded from the review study were other interRAI assessment suites and studies that did not meet the inclusion criteria. The date of retrieval was removed to allow for a more comprehensive search summary and a complete output from the databases.

**Table 2.** Keywords Description.

Key term	Description	Search terms and synonyms
Care planning	Care planning consists of negotiations and agreements between care providers and residents to develop relevant health plans (care plans) throughout the interrelated processes of performing health assessments, formulating care plans, and implementing and evaluating the care provided (Burt et al., 2014).	activit” OR “implement” OR “plan” OR “goal” OR “Patient Care Plans” OR “Health and Welfare Planning” OR “Treatment Planning” OR “Caring Behaviors” OR “Discharge Planning” OR “Post-treatment Follow-up” OR “Case Management” OR “Discharge Planning”
		AND
Long-term care	Long-term care facilities that provide 24-hour support are often called nursing homes, long-term care, residential care left or seniors’ residences (Macdonald et al., 2020)	“long-term care” OR “nursing home” OR “care home” OR “residential” OR “extended W2 care” OR “long-term care”)
		AND
interRAI	The interRAI is a data-driven application that nurses and other clinicians use to collect clinical data upon a resident’s admission, and quarterly and annually, to generate plans that inform a particular LTC resident’s care (Armstrong et al., 2017; Hutchinson et al., 2010).	“interrai”

### Study Selection

**Figure 1.**
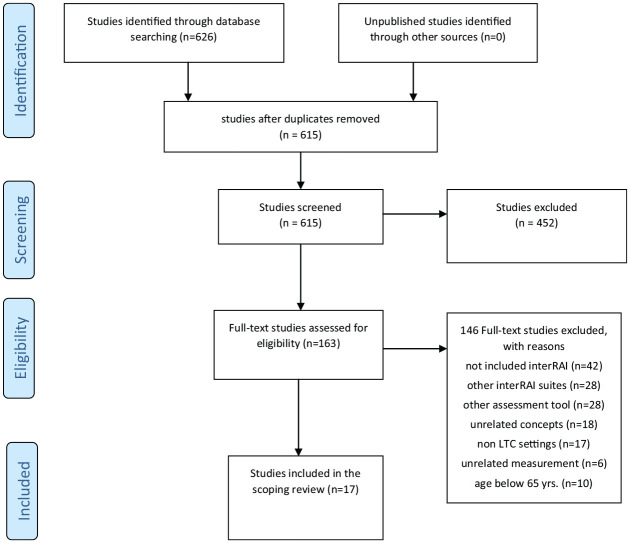
PRISMA flow diagram of the scoping review process.

In total, 626 studies were retrieved and uploaded into Covidence software (Veritas Health Innovation, Melbourne, Australia), and 11 duplicates were removed. Two reviewers from the team screened the titles and abstracts of 615 studies, and 452 irrelevant studies were excluded. A total of 163 selected full-text studies were assessed and screened against the inclusion criteria, of which 146 studies were removed because 42 of these studies did not include the use of interRAI, 28 studies used suites other than interRAI-LTCF, 25 used other assessment tools, 18 included unrelated study concepts, 17 used settings whose purpose was not long-term care, 6 used unrelated measurements, and 10 used participants under 65 years of age. Included in this review were 17 studies that met the eligibility and inclusion criteria. Figure 1 presents a flow diagram of the search results, in accordance with the Preferred Reporting Items for Systematic Reviews and Meta-Analyses extension for Scoping Reviews (PRISMA-ScR) (Tricco et al., 2018).

### Quality Appraisal

Two reviewers performed quality assessments of the articles using JBI’s appraisal checklist (Joanna Briggs Institute, The University of Adelaide. n.d.) and the 2018 version of a mixed-methods appraisal tool (MMAT) for quantitative and mixed-methods studies (Hong et al., 2018). Although it is not compulsory to perform quality assessments of the studies searched when conducting a scoping review, if this type of review is to inform research, policy, and practice, then a quality assessment will provide a panoramic and intellectual overview of what is known and of the noteworthy knowledge gaps in the literature (Davis et al., 2009). Therefore, we verified the quality of the evidence provided in the studies as it strengthened the applicability of our results. Moreover, each item on the JBI checklist received a score that ranged from 0 (poor quality) to 2 (high quality), and the MMAT 2018 version also received the same scores as the JBI-appraised items. A total quality score was calculated by adding all of the item scores of the appraised tools. Any score of less than 13 for the JBI-appraised articles and scores of less than 7 for the MMAT 2018 version were considered poor-quality studies. However, all of the studies included for this scoping review exceeded the average scores for a quality assessment (Table 3).

**Table 3.** Characteristics of included studies.

Authors/country	Research aim	Methods	Participants/sample	Key findings based on interRAI utility in care processes	Quality appraisal JBI* MMAT*
Achterberg et al. (2010) Finland, Italy, and the Netherlands	To document the prevalence of pain, its frequency and severity as well as its correlates in three European countries.	Cross-sectional study	Patient aged 65 and above comprised 5,761 from 64 facilities in Finland, 2,295 patients from eight facilities in the Netherlands and 1,959 patients from 31 facilities in Italy.	Clinical data collection and repository* Minimum dataset of interRAI was used to gather relevant clinical data of the residents for assessment. Based on data comparison and analysis, the authors concluded that pain is frequently encountered in long-term care facilities in Europe and that, despite cultural and case-mix differences.	13
Blekken et al. (2016) Norway	To explore prevalence, associations, and effect of clustering of observation units and variation in fecal incontinence among nursing home patients.	Cross-sectional study	A total of 261 patients from 20 nursing home units between September–October 2014	The Norwegian version of the interRAI was used to collect clinical data from and mixed-effect models’ analysis of interRAI data collected shows prevalence of fecal incontinence was 42·1% or 54%. The effect of clustering by nursing home unit was not statistically significant. variation in fecal incontinence rates was explained by differences in patient characteristics.	14
Foebel et al. (2015) Canada	The study investigates the frequency and correlates of new antipsychotic (AP) drug use among newly admitted LTC residents.	A retrospective, longitudinal study	The sum of 47,768 retrospective residents’ data was collected with interRAI instrument by trained nurses.	The researchers analyzed residents’ data (clinical, social, demographic, and medication use) collected between 2003 and 2011. The results indicate that new AP drug users comprised 7% of the final cohort. Severe cognitive impairment, dementia, and motor agitation were significantly associated with new AP drug use among both sexes.	15
Kehyayan et al. (2016) Canada	To identify predictors of long-term-care (LTC) facility residents’ self-reported quality of life (QoL).	Secondary data analysis	Convenience sample of 928 residents from 48 volunteer LTC facilities across six Canadian provinces.	Analysis of residents’ interRAI Self-Report Nursing Home Quality of Life survey data indicates that QoL is significantly associated with select resident and LTC facility characteristics.	14
Lam et al. (2017) Hong Kong	To review the change in the prevalence of physical and chemical restraint in long-term-care facilities (LTCFs).	Observational study	A total of 10 residential LTCFs with 2,896 participants data were retrieved from interRAI between 2005 and 2015	Trained assessors (nurses, social workers, and therapists) collected residents’ clinical data with interRAI tool. Analysis of data collected shows an increasing trend in the use of physical and chemical restraint among LTCF residents in Hong Kong over a period of 11 years.	13
Gindin et al. (2014) Czech Republic, France, Finland, Germany, England, the Netherlands, Italy and one non-European country (Israel)	To assess insomnia and its correlates as part of the Services and Health for Elderly in Long-term care	Cross-sectional study	Elderly residents (*N* = 4,156) of 57 LTCFs in eight European countries and Israel	Health status assessment* The researchers used interRAI scales to assess and record residents’ insomnia, activities of daily living (ADLs), cognitive status, depression, major stressful life events, physical activity, fatigue, pain, sleep medication use and other demographic variables like age and sex was extracted from the tool. Overall, the study shows that hypnosedatives and depression were strong predictors of insomnia beyond cultural differences.	14
Lukas et al. (2013) European countries	To identify pharmacological and nonpharmacological pain management approaches and associated factors in nursing home residents across Europe	Cross-sectional study	A total of 4,156 residents participated in the study across Europe	Residents were assessed using the interRAI instruments (interRAI-LTCF), on pharmacological and nonpharmacological pain management modalities. The findings show that pain treatment in European nursing homes remains to be suboptimal and requires further improvement.	15
Vetrano et al. (2016) Italy	To assess the association of anticholinergic medication burden with hospitalization and mortality in nursing home elderly patient’s disease	Retrospective observational study (5 years)	A total of 3,761 nursing home older residents’ data	A comprehensive clinical and functional assessment was carried out with interRAI instruments. The anticholinergic burden was assessed through the anticholinergic cognitive burden (ACB) scale. The findings show that anticholinergic medication burden is associated to hospitalization and all-cause mortality in institutionalized older adults	13
Vetrano et al. (2018) Czech Republic, Germany, England, Finland, France, Italy, and Netherlands plus Israel	To test the association between polypharmacy and 1-year change in physical and cognitive function among nursing home (NH) residents	Longitudinal multileft cohort study	NH in Europe (*n* = 50) and Israel (*n* = 7). A total of 3,234 older residents participated in the study	Participants were assessed with the interRAI scales, which include the following: Cognitive function was assessed through the cognitive performance scale (CPS). Functional status was evaluated through the Activities of Daily Living (ADL) Hierarchy scale. The change in CPS and ADL score, based on repeated assessments, was the outcome, and their association with polypharmacy was modeled via linear mixed models.	13
Sørbye et al. (2019) Norway	To describe the use of opioids in nursing homes during a 5-year period	Longitudinal study	Participants include palliative residents and nurses (*n* = 100)	interRAI assessment focused on palliative care, symptoms, and suffering during the last 3 days before death. Semi-structured interview was conducted for nurses on duty at the deathbed. The findings show an incremental use of opioids for palliative residents from the first assessment to the time of death.	15
Fedecostante et al. (2020) Czech Republic, Germany, England, Finland, France, Italy, and Netherlands plus Israel	To identify independent predictors of functional decline in older nursing home (NH) residents, considering the resident and facility characteristics	Longitudinal observational study	1,760 older (≥65 y) residents of NH from 57 NH in eight countries	Health-risk prediction* All residents had a comprehensive geriatric assessment using the interRAI-LTCF. The mixed-effect logistic regression model of interRAI assessment shows NH residents experienced ADL decline and facility characteristics also predict the risk of functional decline	14
Hirdes et al. (2014) Canada	The study aimed to predict mortality using interRAI CHESS Scale among persons with neurological conditions in three care settings	Cross-sectional study	Data was retrieved from persons in home care (*n* = 359,940), complex continuing care hospitals/units (*n* = 88,721), and nursing homes (*n* = 185,309) in seven Canadian provinces/territories.	Survival analyses were done with interRAI assessments linked with mortality data. Findings show that CHESS scale was a significant predictor of mortality in all three care settings for the 11 neurological diagnostic groups considered after adjusting for age and sex.	13
Kron et al. (2003) Germany	To identify individual predisposing risk indicators for falls in a sample of institutionalized frail elderly	Prospective observational study	472 long-term-care residents participated in the study with 1-year follow-up	InterRAI fall risk indicators were operationalized in the study. Crude odd ratio calculation for fall indicators shows, of the 46 potential risk indicators considered, 21 seemed strongly predict the risk of falling and/or the risk of experiencing multiple falls	15
Poss et al. (2010) Canada	To develop a bedside MDS-based (interRAI PURS) that will identify individuals under care at various levels of risk for developing pressure ulcers.	Secondary data analysis	Data for developing initial scale included three LTC homes with 257 residents and 89 Ontario LTC homes with 12,896 residents with baseline/reassessment MDS data between 2005 and 2007	interRAI PURS (the new scale) differentiates risk of developing pressure ulcers among facility-based residents by eliminating duplicated effort required for separate pressure ulcer risk scoring.	14
			Data for further baseline/reassessment samples included 13,062 patients of Ontario Complex Continuing Care Hospitals (CCC) and 73,183 Ontario long-stay home-care (HC) clients.		
Williams et al. (2015) Canada	To examine a person-lefted care program implemented in three Canadian long-term care facilities, and to determine its effect on resident health outcomes	Mixed-methods design	There were 682 residents in the Intervention Group (441 women, 241 men) and 512 residents in the Comparison Group (371 women, 141 men).	Care plans and interventions* Using the interRAI scale scores and quality indicators, the researchers retrospectively examined resident outcomes before, after and 6 months following the initiation of the program in both intervention and control groups The finding shows no program effects on resident health outcomes. Facilities approach to care (program and control group) did not show any systematic differences	9
Frijters et al. (2013) Czech Republic, Finland, France, Germany, Italy, Israel, Netherlands, and England	To show how LTC facilities at facility and country level can be compared on quality of care using thresholds and a Quality-Indicator sum measure	Longitudinal design	At baseline data were collected from 4,156 residents, at 6 months follow-up data from 3,761 residents, and at 12 months follow-up from 2,686 residents.	Performance and quality improvement* The researchers enhance the comparison of QIs between facilities and countries by using the method of percentile thresholds and developed a QI sum measure based on percentile outcomes. The findings show that interRAI-LTCF instrument quality of care between LTC facilities in and across nations can be adequately compared.	17
Sales et al. (2011) Canada	To elicit priority rankings of indicators of quality of care among providers and decision-makers in continuing care	Qualitative design (modified nominal group technique)	A total of 47 people representing seven of the nine health regions in Alberta participated in four meetings	Prioritization of interRAI quality indicators and resident/client assessment protocols resulted in the selection of the following quality indicators: The top-ranked items from the long-term-care assessment data were pressure ulcers, pain, and incontinence for the province of Alberta, Canada.	14

* JBI score less than 13 = poor quality.

* MMAT score less than 7 = poor quality.

* interRAI utility in care processes.

### Data Extraction and Synthesis

The extracted data included the authors, year and country of publication, the studies’ aims and methods, the participants and sampling, the specific uses of the interRAI tool in care processes, and assessments of the quality of the reviewed studies.

## Results

The included studies were conducted mainly from within the European Union (EU). Four studies are from European countries with cross-sectional and longitudinal designs, respectively (Blekken et al., 2016; Kron et al., 2003; Sørbye et al., 2019; Vetrano et al., 2016). Two studies are EU multicenter studies with cross-sectional research designs (Achterberg et al., 2010; Lukas et al., 2013). The last four EU studies are multicentered with the inclusion of the State of Israel and their research designs range from cross-sectional to longitudinal, respectively ( Fedecostante et al., 2020; Frijters et al., 2013; Gindin et al., 2014; Vetrano et al., 2018). There are six Canadian studies, two of which include analyses of secondary data (Kehyayan et al., 2016; Poss et al., 2010), one is a cross-sectional design (Hirdes et al., 2014), one uses a retrospective design (Foebel et al., 2015), the last two studies use qualitative and mixed-methods designs, respectively (Sales et al., 2011; Williams et al., 2015). Lastly, there is only one observational study from Hong Kong (Lam et al., 2017). All studies included were conducted between 2010 and 2019.

Of the final 17 studies included in the scoping review, five (29.4%) addressed interRAI’s minimum dataset component as a clinical data-collection tool (Achterberg et al., 2010; Blekken et al., 2016; Foebel et al., 2015; Kehyayan et al., 2016; Lam et al., 2017), five (29.4%) addressed interRAI’s scales and clinical-assessment protocols as health-assessment tools (Gindin et al., 2014; Lukas et al., 2013; Sørbye et al., 2019; Vetrano et al., 2016, 2018), four (23.5%) considered the assessment scales of the interRAI tool in terms of whether it is capable of predicting residents’ health risks (Fedecostante et al., 2020; Hirdes et al., 2014; Kron et al., 2003; Poss et al., 2010), one (5.9%) investigated the effects of interRAI-driven care plans on residents’ health outcomes (Williams et al., 2015); and the remaining two studies (11.8%) used interRAI’s quality-indicator function as a means of measuring care performance and improvements in the quality of care (Frijters et al., 2013; Sales et al., 2011). Based on the reviewed studies, the findings were grouped into five key care processes and health domains or themes: clinical data collection; health assessment; health-risk prediction; care plans and interventions; and care performance and improvements in the quality of care.

### Clinical Data Collection

A minimum dataset (MDS), which is a component of interRAI, was used to collect clinical data from residents. Residents’ subjective clinical data is an essential component of an MDS, as it uses standardized language and data-driven algorithms (Carpenter & Hirdes, 2013). The most significant innovation in the newer version of MDS 3.0, as compared to MDS 2.0, is that it is possible for the assessor to directly interview residents rather than relying on other clinical documentation on these residents (University of California San Francisco (UCSF) Division of Geriatrics, Department of Medicine, 2018).

#### Measures of quality of life

MDS questionnaires comprise different clinically guided or probing questions that assess every area of residents’ physical, cognitive, mental, social, and individual preferences and needs. In a study by Kehyayan et al. (2016), subjective data on residents’ quality of life (QoL) was collected using interRAI’s self-reported QoL survey, which consists of 10 domains that each contains from 4 to 6 items. These QoL domains include privacy, food or meals, safety and security, comfort, autonomy, respect, responsive staff, staff-resident bonding, activities options, and the support and promotion of personal relationships (Kehyayan et al., 2016).

#### Pre-assessment data

The assessors in a study by Achterberg et al. (2010) collected data on residents’ pain by using an MDS of pain-frequency items that were coded as “no pain,” “less than daily pain,” and “daily pain” within the past 7 days, and pain-intensity items that were coded as “no pain” and “mild, moderate, or severe pain” within the past 7 days. Moreover, MDS data not only enhances health assessments but also provides the clinician with relevant knowledge that informs the provision of appropriate interventions for residents. Poss et al. (2010) aggregated MDS data to develop a Braden scale that could identify at-risk residents for pressure ulcers; the intention was to improve the quality of care provided to vulnerable residents. Importantly, clinical information collected with MDS could inform daily care plans for long-term-care residents. For example, constipation and diarrhea are classic side effects of medication intake, especially among residents who have more than one medication intake per day. In a study by Blekken et al. (2016), trained assessors used MDS data on section H3, which tracks bowel incontinence, according to a rating scale of one to eight, to manage residents with daily fecal incontinence.

#### Data-driven management of chronic diseases

Managing psychiatric symptoms in LTC residents can be overwhelming for care providers. Research indicates that more than half of LTC residents may have dementia, depression, psychosis, or other cognitive impairments (Canadian Coalition for Seniors’ Mental Health, 2009). Accessibility to MDS’s big data and the ability to analyze residents’ clinical information for the use of restraints and antipsychotic medications could provide insights into what could benefit resident populations that exhibit mental symptoms and promote their safety and the safety of other residents. For example, a study by Foebel et al. (2015) used baseline data as well as 6 months of MDS data that was collected from LTC residents to compare antipsychotic-medication users and non-users, continuous users, and those who had to discontinue their antipsychotic medications. Their study concluded that behavioural, social, and clinical factors significantly influence new prescriptions of antipsychotic drugs after LTC admission. In another similar study by Lam et al. (2017), trained assessors used a minimum dataset from 10 residential LTCFs to determine the prevalence of the use of physical and chemical restraints on residents with mental health challenges. The implication of the use of an MDS in these studies is that care providers could aggregate, analyze, and monitor residents’ data over a period of time, while also comparing it with the MDS, to improve the provision of care and the allocation of resources for both the residents and the facilities.

### Health Assessment

Based on the information collected from residents through the use of MDS questionnaires, an assessment scale measures a particular domain of the residents’ health statuses. The scales show results based on MDS information that is recorded for care purposes alone (Carpenter & Hirdes, 2013) For instance, once an MDS has been completed online, the underlying algorithms in the interRAI generate assessment scales that provide measures of severity, such as the extent of a resident’s dependency regarding assistance in carrying out activities of daily living (Carpenter & Hirdes, 2013).

#### Multi-dimensional scales

interRAI’s assessment scales are coordinated by algorithms that make it possible for this tool to provide the diagnostic and predictive functions required in the planning and delivery of care. Like any algorithm, interRAI’s capacity to predict a health risk or to correctly capture a decline in a resident’s health status is predicated on the amount of data that is imputed. Sorbye et al. (2019) developed scales that determine the need for opioids use during palliative care and prior to a resident’s death. They include an activity of daily living scale that ranges from 0 to 6 for items like movement, personal hygiene, toileting, and nutrition; a value greater than or equal to 3 indicates that the resident requires comprehensive help. Other scales include the cognitive performance scale (6–9-point scale) to evaluate residents’ memory, where a value greater than or equal to 3 indicates moderate to severe problems. The communication scale (4-point scale) is used to measure residents’ self-understanding and whether others understand them, where a value greater than or equal to 4 shows moderate to severe cognitive impairment. In the same study, residents’ clinical depression status was assessed with a depression rating scale (14-point scale), where a value greater than or equal to 3 indicates depression. The frequency and intensity of pain are assessed on a 5-point scale, and a higher score indicates intense pain. With several scales scoring higher values as resident’s health degenerates at the end of life, these researchers indicated the increased need for opioids for comfort measures and pain management.

#### Propensity for multi-domain assessment

The sequence of events that are listed through the use of an MDS to collect a resident’s health information and generate assessment scales that inform the clinician of the severity of the changes in a resident’s health should support the precise interventions that are needed to help each resident reach a significant level of functioning that is both achievable and tolerable.

Using the same assessment scales as above (activity of daily living scale, cognitive performance scale, and depressive rating scale), Gindin et al. (2014) evaluated the prevalence of insomnia and its correlates among LTC residents and found that hypnosedatives, depression and psychosocial variables predict this insomnia. To assess pain as a correlate to insomnia, Lukas et al. (2013) assessed the pain levels of LTC residents, using interRAI’s pain scale (4-point), where a resident that presents with no pain measures as= 1; pain that is present but not within the past 3 days measures as = 2; pain that is present on 1 to 2 of the past 3 days = 3; and daily pain that presented within the past 3 days measures as = 4. These researchers found that symptoms of pain vary among residents across countries in Europe.

In another study, Vetrano et al. (2016) investigated residents’ functional assessments as measured according to the ADL hierarchy scale (0–6 points), a cognitive performance scale, and a depression rating scale, and found that the use of anticholinergics was associated with functional decline in residents. In 2018, Vetrano et al. (2016) conducted another study that used interRAI’s cognitive performance scale and the ADL hierarchy scale at a baseline and at 3, 6, and 12 months to test the relationships between polypharmacy and post-1-year changes in physical and cognitive function among LTC residents. Their study found that polypharmacy was associated with worsening cognitive function but not with the functional decline among residents.

### Health-Risk Prediction

With the adequate reliability of interRAI’s tool for assessing the health of older adults (Hirdes et al., 2008; Kim et al., 2015), interRAI scales could be used as measures of health outcomes to compare residents’ health over a period of time and determine those who face serious health risks. To predict mortality among residents with neurological conditions, the health services used and the caregiver distress in nursing home populations, Hirdes et al. (2014) used interRAI’s changes in health, end-stage, and signs and symptoms scale (CHESS) and found that the scale’s predictive capability performed consistently well in predicting resident mortality and in care planning and service delivery in LTCFs. Moreover, Kron et al. (2003) used a translated version of interRAI as an operationalized definition and screening tool to measure residents at risk of falling and concluded that urinary incontinence, cognitive impairment, the use of restraints, depression, and transfer difficulties are modifiable predisposing risk factors.

Another serious health concern in LTC is the rapid functional decline of residents over a period of time. As functional decline among institutionalized residents is exacerbated by cognitive decline, hospitalization, and continence decline (Jerez-Roig et al., 2017), predicting those at risk of decline could help care providers by providing appropriate interventions for these residents. For example, in a study by Fedecostante et al. (2020), residents underwent a comprehensive assessment that used multi-item scales that were embedded in the interRAI tool and included 1-year follow-ups to identify what predicts a functional decline in older LTC residents. They found that severe dementia and urinary incontinence are common among LTC resident populations with greater antipsychotics use.

### Care Plans and Interventions

interRAI-driven care plans add residents’ voices or those of their representatives to care-delivery processes. Irrespective of the health issues or concerns the interRAI tool identifies, care providers are obligated to engage the residents, or their care representatives, with these critical issues before deciding on the priority of the care or intervention. The interRAI MDS uses residents’ clinical data to trigger a set of clinical assessment protocols (CAPs) that indicate the problem areas in the residents’ health that their care plans need to address (interRAI, 2021). These CAPs do not automate care planning; however, they help the clinician, the residents or their representatives focus on important issues that are identified during the assessment process so that decisions on how to intervene can be explored from the residents’ perspectives (interRAI, 2021). However, as good as engaging residents in care delivery is, studies show that health outcomes are mixed for long-term-care residents who receive person-centred care (Williams et al., 2015). To prove this assertion, Williams et al. (2015) used scores from several interRAI scales, and a component called a “quality indicator” in three long-term care facilities to analyze the effects of person-centred care on residents’ health outcomes at the baseline, after the interventions were introduced, and again during a follow-up at 6 months. They did not find any significant effects on residents’ health outcomes when person-centred care programs were implemented.

### Performance and Quality Improvement

InterRAI quality indicators (QIs) are components that measure residents’ health performance across several LTCFs. These QIs are derived from aggregated clinical data that measures care quality improvements, at the facility level (Carpenter & Hirdes, 2013), by identifying the areas in a facility’s care provision in which it may be underperforming. Frijters et al. (2013) used the QIs components of the interRAI tool to enhance comparison of facilities’ performance in European Union countries. Using percentile thresholds and QIs’ sum measure to show individual facilities’ performance across several metrics, they found that the interRAI-LTCF instrument facilitates a comparison of the quality of care among LTC facilities in terms of continuing improvements (Frijters et al., 2013). However, Sales et al. (2011) contextualized interRAI’s usefulness quality performance function in long-term-care facilities (LTCFs) by adding the CAPs’ automated assessment function to QIs to prioritize and rank the care components that had the greatest effects on residents’ health outcomes. The top-ranked items from the long-term care assessment data were pressure ulcers, pain, and incontinence (Sales et al. (2011).

## Discussion

Evidence from the studies shows that the interRAI tool provides a viable way of collecting clinical data for assessment, identifying residents with health risks, supporting the formation of appropriate care plans, and improving the quality of care provided to long-term-care residents and the performance of the facilities in which these people reside. However, there is inadequate evidence to support how care plans that are driven by interRAI translate into quality care provision for residents. This gap points to an urgent need that should be explored through a more focused review of the impact of interRAI on care planning within LTC settings. The only literature in this review that addresses the utility of interRAI-driven care plans emphasizes the concept of patient-centred care plans and activities that are empowered not only for the residents themselves and their care representatives but also by the care providers who consider this approach to be a way of retaining residents’ independence and preventing their decline throughout their stays in these facilities (Williams et al., 2015).

The limited evidence that supports the implementation of interRAI care plans for positive health outcomes is consistent with other findings that point to interRAI’s care plan implementation as being impaired by the lack of cohesion that exists between frontline staff and interdisciplinary care teams (Colón-Emeric et al., 2006). Similarly, Kontos et al. (2010) attributed the inadequacy of interRAI care plans in meeting the varying needs of long-term care residents to the tool’s failure to capture or relate personal support workers’ contributions to interdisciplinary care teams. While the lack of cohesion and connection among LTC staff emanates from differences in professional roles within nursing homes (Daly et al., 2002), licensed professionals, such as registered nurses (RNs), often presume that frontline personal support workers (PSWs) lack the educational capacity to implement basic care to their residents (Kontos et al., 2010). Consequently, PSWs are usually excluded from interdisciplinary care-plan teams (Kontos et al., 2010), despite providing 80% to 90% of all direct care in LTC facilities (Caspar & O’Rourke, 2008). In turn, PSWs consider the interRAI tool as irrelevant and this often leads to low or no compliance with directives that come through the use of this tool (Kontos et al., 2010). Evidence also suggests that coordinating care processes among multiple registered nurses (RNs) can improve communication by 50% but it also finds that when one RN coordinates the care-planning activities, there is a 50% decline in the sharing of this information (Adams-Wendling et al., 2008). Consequently, nursing homes often find that their care-plan implementation and residents’ health outcomes significantly differ, despite the use of interRAI to coordinate these homes’ health assessments and care planning (Taunton et al., 2004).

Further, standardized interRAI care plans have failed to consistently result in quality health outcomes for residents in other similar homes (Kontos et al., 2010). Some of the factors implicated in interRAI’s inefficiencies are, first, the fact that long-term care facilities vary in structure, staff, and operational capacity and this often contributes to differences in the overall performance of the care delivery (Bott et al., 2007); and, second, that the care plans do not guide the daily care in LTCFs (Dellefield, 2006; Schnelle et al., 2004) and this results in residents not receiving quality care (Colón-Emeric et al., 2006). It is imperative for care providers to understand that the interRAI tool offers invaluable support to clinical decision-making in coordinating the care of long-term care residents. However, a lack of clarity regarding residents’ preferences limits the instrument’s care-planning and intervention function in terms of meeting residents’ health needs; this results in its decreased usability and poor implementation (Turcotte et al., 2018). For interRAI’s use to result in consistent health outcomes for long-term care residents, care plans need to be explored directly with residents or their families and frontline registered nurses and care aides. Measures of care quality differ from one resident and their family to another. Attempts to formulate residents’ care plans should be guided by what is mutually agreed upon by the residents or their families and the nurses who either direct or deliver this care.

## Strengths and Limitations

The criterion of having two reviewers screened the titles, abstracts, and full texts of the articles against the inclusion and exclusion criteria strengthen the trustworthiness of this study. We did not set a date limit for the literature search, and an inclusion factor required that the articles be written in the English language. While the former collates an enormous number of articles from databases that did not address the review objective, the latter consider non-English language studies a factor that limits the likelihood of the transferability of the results to English-speaking knowledge users and audiences alone.

## Recommendations for Further Research

Since continuous improvements in the quality of care for long-term care residents is an important priority for LTCFs around the world, it is important to understand the various factors that either foster or impede the use of the interRAI tool to devise standardized care plans for LTC residents. The direction of the research on interRAI-driven care plans should be explored with frontline care providers (registered nurses and care aides) and residents in terms of the applicable ways of developing and implementing care plans for the benefit of these residents.

## Conclusions

This review shows that the interRAI digital tool can be successfully used to coordinate residents’ care processes. However, it also indicates that there is inadequate evidence to support the implementation of interRAI-driven care plans for consistent health outcomes. In addition, the increased use of interRAI-driven care plans within LTCFs will require that care providers be committed to continually meeting each resident’s specified needs and preferences.
